# Rebuilding the Marrow *In Vitro*: Translational Advances in the 3D Modeling of Blood Cancers

**DOI:** 10.3390/onco5040051

**Published:** 2025-11-23

**Authors:** Giovannino Silvestri, Aditi Chatterjee

**Affiliations:** 1 Marlene and Stewart Greenebaum Comprehensive Cancer Center, Baltimore, MD 21201, USA; 2 Member Cancer Therapeutics Program, Marlene and Stewart Greenebaum Comprehensive Cancer Center, Baltimore, MD 21201, USA; 3 Department of Medicine, University of Maryland School of Medicine, Baltimore, MD 21201, USA

**Keywords:** organoids, 3D marrow-mimetic systems, AML, CML, bone marrow niche, 3D bioprinting, microfluidics, precision medicine, tumor microenvironment, immunotherapy

## Abstract

Hematological malignancies such as acute myeloid leukemia (AML), chronic myeloid leukemia (CML), lymphomas, and multiple myeloma remain difficult to model *ex vivo* because conventional two-dimensional (2D) cultures and murine systems fail to reproduce the spatial, metabolic, vascular, and immune complexity of human bone marrow and lymphoid niches. Recent advances in three-dimensional (3D) platforms—including spheroids, engineered organoid-like marrow models, and microfluidic niche-on-a-chip systems—now allow for a more physiological replication of stromal, endothelial, and immune interactions that drive resistance and relapse. In this review, we introduce explicit definitions distinguishing spheroids, organoid-like constructs, true hematopoietic organoids, and microfluidic devices to establish a unified framework for hematologic 3D modeling. We synthesize applications across AML, CML, lymphoma, and myeloma, highlighting mechanistic insights, strengths, and limitations unique to each disease. Finally, we outline a translational roadmap that integrates bioprinting, perfusable vasculature, immune reconstitution, and AI-driven analytics toward next-generation patient-specific platforms. These innovations position 3D marrow-mimetic systems as essential tools for precision oncology in blood cancers.

## Simple Summary

Preclinical models for leukemias, lymphomas, and myeloma often miss the complexity of human bone marrow and lymphoid niches that shape drug resistance. New three-dimensional (3D) marrow-mimetic systems—including microphysiological “marrow-on-a-chip,” engineered co-cultures, and early organoid-like platforms—better reproduce stromal, vascular, immune, and metabolic cues. We review how these models are being used across AML, CML, lymphomas, and myeloma, where they excel, where they fall short (e.g., lack of functional vasculature and limited immune competence), and how emerging bioprinting, microfluidics, and AI analytics can accelerate precision medicine.

## 1. Introduction

Hematological malignancies—including acute myeloid leukemia (AML), chronic myeloid leukemia (CML), lymphomas, and multiple myeloma—represent a diverse group of cancers that arise from hematopoietic and immune systems and remain significant contributors to global cancer morbidity and mortality [1,2]. Their biology reflects dysregulated hematopoiesis, clonal expansion of aberrant progenitors, and disruption of stromal and vascular architecture within bone marrow and lymphoid tissues. Genetic, epigenetic, and microenvironmental alterations collectively drive proliferation, immune evasion, and therapeutic resistance, underscoring the need for experimental systems that faithfully capture the complexity of human hematopoietic niches [1–4].

Traditional preclinical platforms—most notably two-dimensional (2D) cultures and murine xenograft models—have provided foundational insights into signaling pathways, clonal evolution, and drug response. However, these systems lack the spatial organization, matrix composition, metabolic gradients, and immune interactions characteristic of the marrow and lymphoid microenvironments [5–9]. Standard monolayer cultures do not reproduce physiologic stromal–hematopoietic interactions or gradients of oxygen, nutrients, and cytokines that regulate stem cell behavior [5,10]. Murine models, while powerful, are limited by species-specific immune, cytokine, and metabolic differences that constrain translational predictability [8].

To overcome these limitations, a new generation of three-dimensional (3D) marrow-mimetic systems is emerging. These platforms—including engineered organoid-like constructs, stromal–endothelial co-cultures, and microfluidic marrow-on-a-chip devices—enable multicellular assembly, physiologic signaling, and mechanical and metabolic conditions that more closely approximate native human niches [5,7,11–13]. They facilitate extended maintenance of leukemic and stromal components under controlled oxygenation, stiffness, and perfusion conditions, offering improved relevance for mechanistic studies and drug testing.

In solid tumor biology, true self-organizing organoids have transformed disease modeling and precision oncology [14–23]. In hematology, however, organoid technology remains in early development. Several pioneering studies have generated bone marrow organoid-like systems and synthetic niche platforms capable of recapitulating sinusoid-like structures, cytokine gradients, and hierarchical differentiation from pluripotent stem cells or patient samples [24–35]. These constructs, while not yet fully self-renewing or autonomous, represent essential transitional steps toward bona fide hematopoietic organoids.

To clarify terminology and avoid conceptual ambiguity, in this review we use “spheroids” to denote simple self-aggregating clusters; “organoid-like” to refer to engineered 3D assemblies incorporating stromal and vascular elements without self-organization; “true hematopoietic organoids” for self-renewing, multilayered structures that remain an unmet goal in AML and CML; and “niche-on-a-chip” for microfluidic devices that model perfusion, shear stress, and spatial gradients. These definitions provide a unified framework for comparing methodologies and for evaluating the translational potential of 3D systems across AML, CML, lymphoma, and myeloma.

## 2. Hematological Malignancies and the Microenvironment

Hematological cancers depend not only on intrinsic mutations but also on their protective stromal and immune niches [1–3]. Within bone marrow, mesenchymal stromal cells (MSCs), endothelial cells, fibroblasts, and immune cells form a specialized microenvironment that supports both normal and malignant hematopoiesis [29,30,36–39]. AML cells exploit this microenvironment to remain quiescent and resistant to cytotoxic agents, sustaining minimal residual disease and relapse [3,9,26]. CML stem cells persist in protective niches even during tyrosine-kinase inhibitor (TKI) therapy, necessitating dual targeting of LSCs and microenvironmental signals [2,3,26]. In lymphomas, interactions between malignant B or T cells and lymphatic stroma shape immune evasion [37–39]. Follicular lymphoma is emblematic: tumor cells induce regulatory T cells (Tregs) and reprogram macrophages into tumor-associated phenotypes [40–43]. Multiple myeloma further exploits the marrow niche by stimulating osteoclast-mediated bone resorption and leveraging IL-6 to enhance drug resistance [29,44]. These interactions modulate responses to immunotherapy [3,45]. Leukemia and lymphoma cells frequently overexpress immune-checkpoint proteins (e.g., PD-L1), suppressing T-cell responses and enabling immune escape [43,46,47]. Checkpoint inhibitors and CAR-T cells have shown success, yet the immunosuppressive milieu remains a challenge [40,41,48–53].

Three-dimensional (3D) marrow-mimetic systems now permit *ex vivo* replication of these cellular and metabolic dynamics, bridging the gap between reductionist cultures and clinical observation [24,54–57]. Organoid-like bone marrow co-cultures support direct visualization of stromal-leukemia crosstalk, quantification of cytokine gradients, and correlation with drug tolerance phenotypes [24–27,45]. Lymphoid spheroids enable immune-checkpoint testing and immune-cell reconstitution, offering a powerful framework for precision immunotherapy studies. Despite these advances, most systems still lack functional vasculature and dynamic immune elements required to fully recapitulate *in vivo* conditions [58–61].

## 3. Methodologies for 3D Marrow Modeling

### 3.1. Organoid-like (Marrow-Mimetic) Systems

Marrow-mimetic 3D systems represent an emerging class of engineered microphysiological platforms designed to replicate the complex cytoarchitecture and functional dynamics of human bone marrow. These constructs are typically generated from pluripotent stem cells (PSCs) or primary patient-derived hematopoietic and stromal cells, embedded within extracellular matrix (ECM) hydrogels such as collagen, Matrigel, fibrin, or synthetic polyethylene glycol (PEG)-based scaffolds [9,11,12,62]. Through careful control of matrix stiffness, biochemical gradients, and cellular composition, these systems aim to reestablish cell–cell and cell–matrix interactions critical to hematopoietic homeostasis, leukemic persistence, and therapeutic response.

The fundamental differences between conventional 2D monolayer systems and emerging 3D hematologic culture models are summarized in Table 1, illustrating how spatial organization, physiologic gradients, and stromal-immune integration enhance translational predictivity.

Architecturally, marrow-mimetic systems have achieved significant progress in recapitulating multicellular heterogeneity and sinusoid-like microarchitecture reminiscent of native marrow [11,14,23,63–65]. They incorporate stromal, endothelial, and hematopoietic populations within spatially defined compartments that allow dynamic cross-talk among niche components.

In hematologic applications, bone marrow organoid-like co-cultures combining hematopoietic progenitors, mesenchymal stromal cells (MSCs), and endothelial cells have been shown to sustain short-term hematopoiesis and maintain AML blast phenotypes with preserved signaling pathways and surface marker expression [24–28,31–35]. Notably, Khan et al. demonstrated the formation of sinusoid-like vascular structures supporting multilineage hematopoiesis and partial recreation of marrow zonation, highlighting the potential of engineered 3D systems to approximate physiologic stem cell niches [24].

Technological convergence has further enhanced the fidelity of these models. The integration of CRISPR/Cas9 gene editing allows selective modulation of stromal or leukemic components to dissect niche-tumor signaling dependencies, while bioprinting technologies introduce spatiotemporal control over stromal patterning, vascular mimicry, and nutrient perfusion [9,59,62,66]. Recent advances in microfluidic design-notably niche-on-a-chip platforms-permit controlled delivery of pharmacologic agents under defined oxygen, shear, and nutrient gradients, closely emulating the mechanical and biochemical microenvironment of marrow sinusoids [58–60,67]. These devices enable functional drug screening focused on niche-driven resistance mechanisms and permit time-resolved imaging of cellular dynamics and therapeutic responses (Table 1) [45,58,68].

Despite these advancements, marrow-mimetic constructs differ fundamentally from true organoids. They are externally assembled rather than self-organizing, lack sustained self-renewal capacity, and often exhibit limited viability and throughput, restricting their use to short-term functional assays and small-scale pharmacologic studies. To transition toward bona fide marrow organoids, future systems must achieve long-term stability, autonomous tissue self-organization, and multilineage hematopoietic differentiation while maintaining genetic and phenotypic fidelity to the patient’s disease state. Achieving these benchmarks would establish a transformative platform for personalized therapy testing and mechanistic modeling of leukemogenesis in AML and CML.

**Figure 1.**
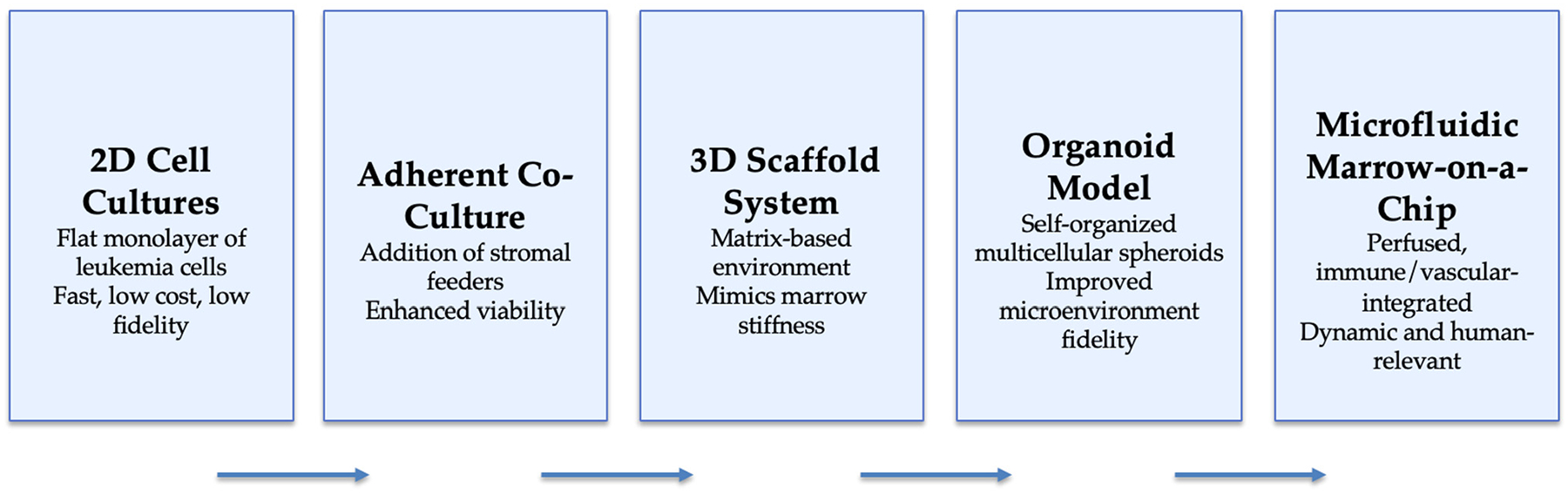
Evolution of hematologic model systems. Schematic representation showing progression from 2D monolayer culture to 3D spheroids, organoid-like constructs, and microfluidic marrow-on-a-chip systems. Physiologic fidelity increases with the incorporation of stromal, vascular, and immune components.

The evolution of hematologic model systems from traditional 2D cultures to advanced marrow-mimetic platforms is summarized in Figure 1, illustrating the progressive integration of stromal, vascular, and immune elements that enhance physiological fidelity and translational relevance.

In this review, we therefore consider organoid-like constructs as critical transitional tools on the path to self-sustaining hematopoietic organoids, with each technological advance (bioprinting, microfluidics, AI analytics) closing specific gaps in physiologic fidelity and predictive power.

### 3.2. Spheroid Models

Spheroid models constitute a more simplified form of 3D culture, offering an accessible yet biologically meaningful platform for modeling cell–cell aggregation, stromal support, and immune interactions in hematologic malignancies. These multicellular aggregates are generated either through spontaneous self-assembly or by seeding tumor and stromal/immune cells in low-adhesion or ultra-low-attachment plates, where cell–cell adhesion molecules drive compact spheroid formation [40,41,54]. Within these structures, nutrient and oxygen gradients develop naturally, enabling exploration of hypoxia-induced signaling, drug penetration, and metabolic adaptation under controlled conditions.

In hematologic applications, spheroids have found the greatest utility in lymphoid malignancies and multiple myeloma, where tumor cells inherently form clusters with stromal and immune partners, mimicking the tumor-microenvironment unit found in lymph nodes or bone marrow [40,41,54]. These models are particularly valuable for drug sensitivity testing, immunotherapy evaluation, and cell–cell interaction studies, as they preserve immune checkpoint signaling, adhesion molecule expression, and cytokine feedback loops. Importantly, patient-derived lymphoma spheroids and organoid-like co-cultures have successfully captured immune-stromal architecture, supporting checkpoint inhibitor and CAR-T testing, thereby extending the relevance of 3D spheroids beyond solid tumor models to the hematologic field [40,41].

In contrast, validated spheroid systems for AML or CML remain unavailable. The intrinsic differences in cellular cohesion, non-adherent growth patterns, and dependence on specialized microenvironments limit the ability of myeloid blasts or stem/progenitor cells to form stable spheroids in isolation. In CML, efforts have instead focused on *ex vivo* expansion platforms for leukemic stem cells (LSCs), emphasizing stromal dependency and cytokine modulation rather than geometric aggregation [19]. Consequently, while spheroids have contributed valuable insights into lymphoid malignancy biology, they are currently insufficient to replicate the complex stem cell niches and hierarchical structure characteristic of myeloid leukemias.

Continued optimization—through incorporation of patient-derived stromal cells, dynamic culture conditions, and immune reconstitution—may extend their relevance to myeloid diseases. Ultimately, hybrid approaches that merge spheroid simplicity with microfluidic precision will yield tractable yet physiologically accurate platforms for translational testing.

A detailed overview of suspension, adherent, scaffold-based, organoid, and microfluidic approaches used to culture AML and other hematologic malignancies is presented in Table 2, outlining representative setups, advantages, and current limitations.

Summary of experimental model types used for acute myeloid leukemia (AML) and other hematologic malignancies, arranged along the continuum from simple suspension cultures to advanced perfused microfluidic systems. Each approach differs in physiologic relevance, technical complexity, and translational potential. Suspension and adherent co-cultures offer rapid, high-throughput screening but limited spatial and microenvironmental fidelity, whereas scaffold-based and organoid systems better replicate extracellular matrix architecture, oxygen and cytokine gradients, and stromal crosstalk. Microfluidic and marrow-on-a-chip platforms provide dynamic perfusion, vascularization, and multi-cell integration, enabling mechanistic modeling of leukemic stem cell behavior, drug resistance, and patient-specific therapeutic responses.

To guide the reader from method to application, the next section (Section 4) is structured to relate each model type to specific disease contexts and to highlight complementarity rather than redundancy among technologies.

## 4. Applications in Hematological Malignancies

### 4.1. AML and CML Research

While organoid technology has transformed modeling in solid tumors, its translation to hematologic malignancies is still emerging.

In AML, a few pioneering studies have created bone marrow-mimetic 3D systems-hydrogels, bone marrow-on-a-chip, and engineered co-cultures-that reproduce oxygen gradients, stromal interactions, and matrix stiffness [58,59,69–73]. These systems (with mesenchymal/endothelial components) show quiescence and increased chemoresistance relative to 2D [5,9,62,69,72,73]. They are not yet true organoids but are critical transitional models toward patient-derived 3D microenvironments.

Maintaining primary AML cells *ex vivo* remains technically challenging due to their limited proliferative capacity and dependence on niche-derived cytokines such as SCF, IL-3, and FLT3 ligand [69,74,75]. In standard media, leukemic blasts rapidly undergo apoptosis and lose clonogenic potential within days. Stromal co-culture or feeder layers can partially sustain survival but often alter gene-expression signatures and promote differentiation, diminishing leukemic stem cell fidelity. These limitations underscore the necessity of 3D marrow-mimetic scaffolds that provide mechanical and biochemical cues essential for long-term LSC maintenance.

In CML, no bona fide organoid/spheroid models have been reported. Instead, *ex vivo* LSC expansion assays maintain phenotypic/transcriptional LSC features and enable preclinical drug screens [19], while microfluidic/hydrogel co-cultures emulate niche signals and LSC persistence under TKIs [2,58,73,76,77]. These approaches emphasize dual targeting of LSCs and the supportive microenvironment.

Leukemia 3D modeling (ALL/AML feasibility). Culturing non-solid leukemia cells from patients *in vitro* remains challenging; however, bone marrow organoid-like and niche-on-a-chip systems have made it increasingly feasible to maintain and interrogate malignant lymphoid and myeloid cells *ex vivo*, including short-term maintenance of ALL/AML blasts within engineered marrow contexts [24–28]. Complementary leukemia-on-a-chip approaches in hematologic disease further demonstrate how bone marrow niche cues drive chemoresistance and can be leveraged for mechanism-guided testing [73]. For CNS-associated leukemias, brain-organoid platforms (as generalizable, tissue-context models) have enabled co-culture paradigms that help dissect signaling pathways relevant to central nervous system involvement and treatment response, illustrating how organoid systems can model multisystem interactions in hematologic malignancies [64,65,78].

Mechanistically, these marrow-mimetic systems have begun to reproduce hallmarks of leukemic stem cell biology that 2D and murine models cannot. Absence of perfused vasculature leads to artificial oxygenation that fails to model LSC hypoxic quiescence, while incomplete immune integration prevents evaluation of T-cell exhaustion, NK surveillance, and checkpoint ligand dynamics central to relapse. Incorporating endothelial lanes and immune modules has revealed how nutrient and oxygen gradients shape FLT3/STAT5 activity, metabolic adaptation, and drug response. Additionally, matrix stiffness and integrin signaling have been directly linked to chemoresistance through MAPK and PI3K activation [54,75,79]. These niche-driven adaptations complement combination strategies targeting FLT3 and cooperating pathways in FLT3-mutated AML [80,81]. Together these mechanistic insights underscore the translational value of 3D systems in uncovering niche-governed drug resistance.

Translationally, microfluidic and bioreactor-based AML/CML chips now allow dynamic dosing under physiologic flow, enabling pharmacokinetic and pharmacodynamic studies in near-real time. Artificial intelligence (AI) integration is increasingly applied to normalize heterogeneity and identify predictive signatures from high-dimensional datasets, connecting mechanistic assays to clinical outcomes.

### 4.2. Lymphoma and Myeloma Research

#### Lymphoma.

Lymphomas comprise a heterogeneous group of malignancies arising from lymphoid progenitors and mature B or T cells, encompassing entities with distinct genetic drivers, differentiation states, and immune–microenvironmental dependencies. Despite their diversity, a common feature across lymphoma subtypes is their intimate coevolution with immune and stromal components of the microenvironment, which shape both tumor biology and therapeutic outcomes. However, there remains a relative paucity of *ex vivo* lymphoma models that fully reproduce the spatial, immunologic, and transcriptional heterogeneity characteristic of these tumors [40,41,45]. Conventional 2D cultures and murine xenografts have limited ability to model the dynamic immune synapses, stromal cytokine networks, and matrix-driven signaling cues that govern lymphoma behavior, creating a gap between preclinical discovery and patient response.

Recent advances in tumor organoid and engineered 3D co-culture systems have begun to address this challenge, offering a means to replicate the immune-rich tumor microenvironment of lymphomas with improved physiological relevance. Notably, these systems have reinforced the central role of B-cell receptor (BCR) signaling as a key determinant of diffuse large B-cell lymphoma (DLBCL) pathobiology, providing experimental evidence that microenvironmental and immune interactions directly modulate intracellular oncogenic signaling [43]. Studies have shown that T cells within the lymphoma microenvironment can influence tumor cell epigenetic landscapes, including the trimethylation of histone H3 at lysine 9 (H3K9me3), thereby altering BCR signaling efficiency, transcriptional plasticity, and therapeutic response [43]. These findings underscore the concept that lymphoma progression is not solely tumor-intrinsic but reflects a reciprocal, microenvironment-driven reprogramming of malignant and immune compartments.

Beyond DLBCL, microenvironment-dependent regulation of gene expression and signaling has been demonstrated across additional organoid and 3D lymphoma systems, including those derived from follicular and mantle cell lymphoma [47]. In these models, extrinsic cues—such as stromal contact, hypoxia, and integrin-mediated adhesion—modulate transcriptional responses and attenuate the efficacy of targeted inhibitors, such as BTK, PI3K, and BCL2 pathway antagonists. This contextual drug resistance reflects physiologic survival mechanisms that are lost in conventional suspension cultures, emphasizing the need for 3D approaches in preclinical drug screening.

Parallel advances in platform development have accelerated the creation of physiologically faithful lymphoma models. Patient-derived lymphoma spheroids, which incorporate malignant B cells with autologous T cells, macrophages, and stromal elements, now recapitulate key immune and stromal interactions observed in follicular lymphoma (FL) [40]. These spheroids exhibit immune checkpoint expression, cytokine exchange, and T-cell activation dynamics, making them ideal for testing immunotherapies and microenvironment-modulating agents that rely on intact immune crosstalk. Similarly, human lymphoma organoids have been engineered to sustain long-term T-cell–tumor interactions, providing an experimental framework to manipulate immune engagement and evaluate mechanisms of T-cell exhaustion, resistance, and rejuvenation under therapeutic pressure [41]. These systems are critical for testing CAR-T constructs, bispecific antibodies, and checkpoint inhibitors, under conditions that closely mimic human lymphoid architecture.

At the structural level, the extracellular matrix (ECM) has emerged as an active regulator of lymphoma biology. Advanced integrin-specific hydrogel systems now serve as adaptable 3D scaffolds for both B- and T-cell lymphomas, enabling controlled interrogation of integrin signaling, transduction, and matrix stiffness effects on proliferation, survival, and drug response [54]. These matrices reveal how microenvironmental mechanics and adhesion-mediated signaling converge with intracellular oncogenic pathways, further linking physical context to functional phenotype.

Collectively, these evolving 3D and organoid-based lymphoma models have begun to redefine how we study tumor–microenvironment co-dependence, immune surveillance, and drug resistance in lymphoid malignancies. By providing immune-competent, physiologically structured, and patient-specific platforms, they enable systematic exploration of how immune dynamics shape therapeutic efficacy. The ongoing development of larger, standardized *ex vivo* resources, integrating genomic, epigenetic, and phenotypic profiling, promises to expand lymphoma drug discovery and immunotherapy modeling [40,41,43,47,54]. Together, these innovations are establishing a new preclinical paradigm—one in which the microenvironment is not a confounder, but a central determinant of therapeutic success.

Remaining challenges include limited vascularization, batch-to-batch ECM variability, and non-uniform immune cell activation across systems. Standardizing stromal-to-tumor ratios and immune inclusion protocols will be key to ensuring reproducibility across laboratories.

#### Multiple myeloma.

Multiple myeloma (MM) is a hematologic malignancy of terminally differentiated B cells (plasma cells) that predominantly colonizes and proliferates within the bone marrow niche, exploiting its cellular and extracellular architecture for survival. Within this microenvironment, complex bidirectional interactions between malignant plasma cells, stromal fibroblasts, osteoclasts, osteoblasts, and immune cells orchestrate a dynamic ecosystem that promotes immune evasion, angiogenesis, osteolytic remodeling, and therapeutic resistance. The marrow niche supplies pro-survival cues via cytokines such as IL-6, SDF-1, and BAFF, while integrin-mediated adhesion and hypoxia reinforce anti-apoptotic and drug-resistant phenotypes characteristic of myeloma persistence.

Conventional two-dimensional (2D) culture systems and legacy myeloma cell lines, though instrumental in delineating key signaling pathways, fail to capture these intricate cell–cell and cell–matrix interactions, mechanical constraints, and oxygen and nutrient gradients that define *in vivo* marrow physiology. This limitation underscores the growing value of 3D marrow-mimetic organoid-like models and microfluidic platforms as next-generation tools for studying myeloma biology and therapeutic response [42,44,45,58,76]. These 3D systems provide a scaffold that sustains plasma–stromal co-dependence, maintains disease-specific heterogeneity, and preserves spatial features of the osteolytic microenvironment, including osteoclast activation and bone resorption signaling.

Recent studies using bone marrow organoid-like cultures derived from patient biopsies have successfully reproduced patient-specific growth patterns and microenvironmental dependencies. Notably, such models have been used to dissect epigenetic mechanisms of pathogenesis, including the role of histone demethylase LSD1/KDM1A, whose dysregulated activity contributes to aberrant transcriptional programs and targetable vulnerabilities in myeloma [42]. The adaptability of these systems also facilitates functional genomic perturbation, drug resistance modeling, and longitudinal monitoring of tumor evolution under treatment pressure.

From a translational perspective, 3D organoid-like and microfluidic models enable high-throughput pharmacologic screening and combinatorial drug testing under near-physiologic conditions, capturing diffusion barriers, stromal shielding, and metabolic gradients that influence therapeutic efficacy [9,58,60,62,67]. The inclusion of vascular components and dynamic perfusion in these systems further enhances drug delivery kinetics, allowing evaluation of penetration efficiency, osteoclast and osteoblast activation, and niche-mediated resistance mechanisms that are otherwise obscured in static cultures [9,58,60,62,67]. These platforms are increasingly leveraged to test novel immunotherapies, including bispecific antibodies, CAR-T constructs, and checkpoint inhibitors, within a microenvironment that more accurately reflects immune suppression and physical confinement of the myeloma niche.

Integration of bioprinting and microfluidic perfusion is accelerating progress toward patient-tailored applications. Modular bioreactors can now co-culture tumor, stromal, and immune compartments with independent control of oxygenation and cytokine gradients, thereby enabling systematic interrogation of therapy resistance and clonal heterogeneity. As these technologies mature, they are expected to support *ex vivo* testing of individualized drug regimens, predictive modeling of response, and discovery of microenvironment-targeted agents capable of disrupting the supportive marrow niche.

Collectively, these innovations are refining our mechanistic understanding of myeloma pathophysiology and providing powerful tools for personalized therapeutic optimization (Table 3) [42,44,45,58,76]. Through the convergence of bioengineering, genomics, and computational modeling, marrow-mimetic myeloma systems are evolving into indispensable platforms that bridge preclinical research and clinical translation, offering a window into disease evolution and treatment resistance in the native marrow context.

Despite these gains, the absence of standardized vascular integration and inconsistent osteoblast–osteoclast dynamics across models continue to limit physiologic accuracy. Cross-validation with patient bone imaging and serum biomarkers may help align *in vitro* remodeling signatures with clinical bone disease parameters.

### 4.3. Cross-Disease Synthesis

Across hematologic malignancies, distinct 3D modeling strategies have matured unevenly but are now beginning to converge conceptually and technologically. AML and CML systems have advanced the mechanistic understanding of stromal adhesion, hypoxia-driven metabolic adaptation, and cytokine-mediated chemoresistance [2,26,58,59,62,69–75,79,82]. In contrast, lymphoma and myeloma models lead the field in immune competence and have demonstrated how autologous immune-stromal crosstalk dictates therapeutic response and immune checkpoint sensitivity [40–44,47,54]. Together, these efforts delineate a functional continuum—from stromal-centric myeloid constructs to immune-dominant lymphoid systems—that can now inform hybridized, physiologically faithful marrow analogs.

In practical terms, the next generation of “integrated hematopoietic organoids” will merge perfused marrow-on-a-chip modules that sustain myeloid stem cell hierarchies with immune-replete lymphoma spheroids capable of real-time T- and NK-cell surveillance [40,41,48,49,58,60,61,67,85]. Such chimeric systems will enable simultaneous study of angiogenesis, immune exhaustion, and metabolic flux within a single controllable microenvironment—approximating the full marrow ecosystem more closely than any current model.

Moreover, the incorporation of endothelialized channels and immune compartments provides a route to test immune-metabolic interactions that are increasingly recognized as central to therapeutic resistance across diseases [2–4,29,40,41]. For example, myeloid models that replicate hypoxic gradients can be connected via microfluidic interfaces to lymphoid modules enriched with T-cell populations, thereby modeling cytokine gradients and immunometabolic feedback in mixed-lineage leukemias and post-transplant immune reconstitution. Early prototypes of these immune-vascularized chips have already shown that perfusion enhances drug penetration and maintains stromal viability for weeks [58–60,67].

Cross-disease integration also extends to computational modeling. Machine-learning frameworks trained on combined AML, lymphoma, and myeloma datasets are beginning to reveal shared gene-expression and signaling signatures of microenvironment-mediated resistance [45,68]. These AI-based analyses complement physical co-culture models by normalizing experimental heterogeneity and identifying conserved pathways—such as PI3K-AKT, MAPK, and SPHK1/S1P signaling—that transcend disease boundaries and predict responsiveness to both targeted inhibitors and immunotherapies [2,3,45,54,79].

Ultimately, hybrid 3D systems that integrate vascular, stromal, and immune components are expected to bridge mechanistic discovery with precision oncology. By combining the microenvironmental authenticity of AML/CML organoid-like models with the immune richness of lymphoma and myeloma spheroids, researchers can create universal marrow surrogates capable of predicting patient-specific therapeutic outcomes, guiding rational drug combinations, and refining clinical trial design [4,40,41,44,45,58–60,62,67]. Such convergence marks the transition from disease-isolated modeling toward a unified, systems-level representation of hematopoietic malignancy.

## 5. Personalized Medicine and Drug Screening

Patient-derived 3D hematologic models are increasingly integrated into precision medicine workflows, particularly within lymphoma and myeloma research, where patient-derived spheroids and organoids have demonstrated predictive validity for therapeutic response [40,41,44,45,86]. These platforms allow *ex vivo* assessment of drug efficacy in conditions that mimic the native bone marrow architecture, supporting the identification of resistance mechanisms and informing individualized therapeutic strategies. In contrast, for acute and chronic myeloid leukemias (AML/CML), fully validated patient-derived organoids (PDOs) remain elusive. Instead, researchers rely on marrow-mimetic 3D systems, including co-cultures with mesenchymal stromal cells, endothelial networks, and niche-like scaffolds, to maintain leukemic stem cells (LSCs) *ex vivo* and evaluate treatment responses [13,19,24–28,45,58,59,68,76]. These systems capture microenvironmental cues-such as hypoxia, stromal-derived cytokines, and extracellular matrix (ECM) signaling-that critically influence disease persistence and drug resistance. Similar organoid-based precision platforms in solid tumors and mixed organoid–immune co-culture systems have already demonstrated predictive value for immunotherapy response and drug screening [87–89].

Technological advancements, such as bioreactor-based perfusion systems and microfluidic organ-on-chip platforms, now permit controlled gradients of oxygen, nutrients, and drugs, enhancing reproducibility and physiologic fidelity [58–60,67]. This dynamic perfusion improves cellular survival, differentiation, and drug penetration, particularly relevant for modeling chemoresistance in hypoxic marrow niches. Furthermore, artificial intelligence (AI)-assisted analysis is being integrated into high-content drug screening pipelines to prioritize effective drug combinations from large datasets, identify synergistic interactions, and correlate phenotypic outcomes with genomic signatures [68]. Collectively, these innovations are advancing the transition from descriptive 3D cultures toward functional *ex vivo* precision platforms capable of informing patient-specific treatment decisions in hematologic malignancies.

Importantly, AI and machine-learning tools also offer a solution to one of the field’s major pain points—variability. By normalizing batch effects and integrating multi-omic data (transcriptomic, proteomic, metabolomic), AI models can uncover consistent predictive markers across diverse 3D systems. Such analytics transform complex imaging and molecular data into actionable therapeutic predictions, ensuring that 3D assays not only mimic biology but also yield reproducible, clinically relevant endpoints.

## 6. Challenges and Limitations

Despite major progress, several technical and biological limitations hinder the widespread adoption of 3D marrow-mimetic systems in translational hematology. A major constraint remains the lack of functional vasculature in most models, which limits nutrient and oxygen diffusion and constrains long-term culture viability [7,13,45,68]. Incomplete immune system integration also impedes the ability to study immune-tumor cross-talk, which is essential for evaluating immunotherapeutics. Moreover, heterogeneity in ECM composition, cytokine supplementation, and stromal-to-leukemic cell ratios across laboratories introduces significant variability and complicates data reproducibility [7,13,45,68].

Recent efforts have introduced endothelialized microfluidic chips capable of generating perfusable vascular networks that improve oxygenation, nutrient distribution, and drug delivery to embedded hematologic cells [59–61,67]. These microengineered systems enhance the physiological relevance of leukemia-on-chip models, particularly for investigating angiogenesis, drug permeability, and pharmacokinetic parameters. Another important advancement is the co-culture of 3D systems with patient-derived immune cells, including T and NK cells, which enables preclinical testing of checkpoint inhibitors and CAR-T therapies under near-physiologic conditions [40,41,48,49]. However, standardization in immune cell sourcing, activation protocols, and temporal integration remains a pressing need.

The absence of sustained vascular perfusion not only restricts culture longevity but also disrupts physiologic gradients critical for modeling leukemic stem cell (LSC) quiescence and hypoxia-driven chemoresistance [2,58,69,72]. Similarly, insufficient immune inclusion prevents assessment of T-cell exhaustion, macrophage polarization, and cytokine feedback loops that define relapse behavior [40,41,43,47]. These mechanistic consequences directly influence drug delivery kinetics and signaling fidelity, emphasizing why functional vascular–immune modules are not optional but essential for realistic disease modeling.

Another unresolved issue is variability across laboratories and manufacturing platforms. Differences in ECM source (e.g., Matrigel vs. collagen vs. PEG-based), matrix stiffness, and cytokine cocktails yield divergent results, complicating reproducibility and meta-analysis [13,59,68]. Establishing standardized reference materials and quantitative metrics such as diffusion coefficients, oxygen tension, and mechanical elasticity, will be essential to allow benchmarking across systems. Furthermore, the absence of regulatory-grade quality control pipelines for 3D biomaterials currently limits clinical translation and FDA validation.

Cost and complexity represent additional barriers. The maintenance of microfluidic or bioreactor-based systems often requires specialized expertise and equipment, limiting scalability for routine use. Nonetheless, the emergence of automated and modular bioreactors—which enable parallelized culture, real-time monitoring, and reduced reagent costs—is progressively mitigating these issues [59,60,67]. Achieving balance between technological sophistication and accessibility will be key to ensuring widespread implementation in both academic and clinical research settings.

Culture medium composition remains a major determinant of model fidelity. Defined, serum-free media offer improved reproducibility and mechanistic clarity by minimizing undefined growth factors, yet they often fail to replicate the complex paracrine and metabolic cues present *in vivo*. Conversely, serum-supplemented or stromal-supported systems enhance short-term cell survival and drug tolerance but introduce batch variability and obscure direct drug response mechanisms [5,7,13,59]. Achieving an optimal balance between physiological relevance and experimental control remains an active area of methodological refinement.

Finally, biological drift remains an underappreciated challenge. Even patient-derived 3D cultures may undergo genetic and phenotypic drift after extended passage, leading to altered clonal composition and signaling heterogeneity. Integration of single-cell sequencing and longitudinal imaging could serve as internal quality control metrics to ensure model fidelity [4,45,68]. Collectively, addressing these limitations will transform 3D marrow-mimetic constructs from descriptive prototypes into standardized, predictive clinical tools.

## 7. Future Directions

The next generation of marrow-mimetic platforms aims to achieve fully vascularized, immune-competent, and multi-organ integrated systems capable of recapitulating complex hematopoietic and leukemic dynamics [7,59–61,85]. Progress in microvascular bioengineering and 3D bioprinting is paving the way toward perfusable capillary networks that support sustained oxygen and metabolite exchange while maintaining marrow-like gradients. Organ-on-a-chip technologies are being designed to interconnect bone marrow analogs with secondary organs, such as the liver and gut, to simulate systemic pharmacology, metabolism, and toxicity-offering a physiologically integrated platform for preclinical drug testing [58,60,67,90].

The development of immune-vascularized marrow chips, capable of supporting autologous T-cell infiltration and cytotoxicity assays, will revolutionize the study of immunotherapy responsiveness and tumor immune evasion [41,48–53,61,85]. These systems will permit real-time imaging of immune–tumor interactions and facilitate optimization of adoptive cellular therapies, including CAR-T and bispecific antibody constructs. Machine-learning integration with multi-omics datasets—combining transcriptomic, proteomic, and metabolomic profiles—will enhance predictive modeling and therapeutic stratification [4,68].

Future studies should also explore the integration of biomechanical and metabolic sensors directly into 3D bioreactors to capture temporal changes in oxygen tension, pH, and metabolite flux. Such real-time analytics will enable adaptive culture systems that automatically adjust perfusion, nutrient supply, and drug delivery according to cellular demand [58,59]. Moreover, linking these data streams to AI-driven feedback control could generate self-optimizing organoid systems capable of maintaining homeostasis over extended periods.

Beyond technological improvements, the field must also establish translational pipelines. This includes harmonized biobanks of patient-derived 3D cultures, ring trial proficiency programs across institutions, and shared reference datasets for machine-learning training [9,45,91]. Ethical and regulatory frameworks will need to evolve in parallel, addressing consent, data sharing, and intellectual property issues surrounding patient-specific organoid models.

At the translational level, establishing harmonized biobanks of patient-derived 3D cultures, coupled with ring trial proficiency programs, will be critical to ensure inter-laboratory reproducibility and validation of results [9,45,91]. The convergence of bioengineering, computational modeling, and systems biology is expected to yield clinically actionable 3D hematologic models—a transformative step toward replacing xenograft testing and enabling real-time, patient-tailored precision oncology.

In summary, the field of 3D hematologic modeling is entering a phase of translation rather than invention. Through the coordinated integration of vascular, immune, and computational modules, marrow-mimetic systems are poised to become indispensable instruments for mechanistic discovery, drug development, and personalized therapy selection. Bridging engineering precision with biological complexity will define the next decade of innovation in blood-cancer research.

## 8. Conclusions

Three-dimensional (3D) marrow-mimetic technologies are transforming the study of hematologic malignancies by bridging the gap between reductionist 2D cultures and the complexity of human bone marrow and lymphoid microenvironments. Organoid-like constructs, stromal–endothelial co-cultures, and microfluidic marrow-on-a-chip platforms now permit physiologic modeling of stromal adhesion, hypoxic gradients, vascular cues, immune interactions, and niche-driven drug resistance in AML, CML, lymphoma, and multiple myeloma. As highlighted across this review, these systems uncover biological mechanisms—such as metabolic adaptation, immune modulation, and LSC persistence—that remain obscured in conventional models.

Despite these advances, major challenges persist, including limited vascularization, incomplete immune integration, variability in extracellular matrix composition, and insufficient standardization across laboratories. Continued innovation in bioprinting, microvascular engineering, immune reconstitution, and sensor-enabled perfusion systems will be essential to achieve long-term, self-sustaining hematopoietic organoids capable of supporting multilineage differentiation and patient-specific disease modeling. The parallel development of harmonized biobanks, ring trial programs, and AI-assisted analytical pipelines will accelerate their clinical translation.

Ultimately, next-generation immune-vascularized marrow analogs have the potential to replace xenograft testing, refine therapeutic decision-making, and support precision oncology workflows across blood cancers. As the field shifts from invention to implementation, integrated 3D systems are poised to become indispensable tools for mechanistic discovery, drug development, and individualized treatment selection in hematologic malignancies.

## Figures and Tables

**Table 1.** Comparison of 2D versus 3D hematologic culture systems. Three-dimensional (3D) organoid and spheroid models recapitulate key physiological features of the bone marrow microenvironment that are absent in conventional two-dimensional (2D) monolayers. These include structural organization, nutrient and oxygen gradients, and integration of stromal, endothelial, and immune components, which collectively enhance translational predictivity for therapeutic response and resistance mechanisms.

Feature	2D Culture	3D Organoids/Spheroids
Cell architecture	Flat monolayer	Spatially organized multicellular structures
Cell-cell interactions	Limited	Extensive, physiologic contacts
Oxygen/nutrient gradients	Uniform	Physiologic gradients (hypoxia zones)
Stromal and immune integration	Minimal	Incorporates stromal, endothelial, immune cells
Genetic stability	Often drifts with passages	Preserves patient-specific clonal diversity
Drug response predictivity	Low to moderate	High, correlates with clinical outcomes
Throughput	High	Increasing with automation and microfluidics
Translational relevance	Limited	Strong correlation with patient response

**Table 2.** Overview of AML and Hematologic 2D and 3D Culture Methodologies.

Model Type	Representative Configuration	Key Components	Advantages	Limitations	Translational Utility
2D Suspension Cultures	Leukemia cell lines (MOLM-14, MV4–11) in serum/defined media	Leukemic blasts only	Easy, inexpensive, high throughput	Rapid apoptosis of primary AML cells; loss of stemness	Drug screening; signaling assays
2D Adherent Co-Cultures	AML cells on MSC or endothelial feeders	Stromal layer + AML blasts	Improves viability; supports cytokine signaling	Feeder variability; non-physiologic architecture	Testing cytokine or adhesion inhibitors
Scaffold-Based 3D Systems	AML + MSC + EC in collagen/Matrigel or PEG hydrogels	ECM scaffold + stromal + hematopoietic	Mimics marrow stiffness, gradient control	Limited vascularization; short-term stability	Mechanistic AML niche modeling
Organoid-Like Cultures	Self-assembled aggregates or bioprinted constructs	AML, MSC, EC, immune cells	Preserves phenotype; supports LSC hierarchy	Technically demanding; limited scalability	Personalized drug testing; mechanistic studies
Microfluidic ‘Marrow-on-a-Chip’	Perfused chips with endothelial and stromal chambers	Stromal + hematopoietic + immune	Dynamic flow; oxygen/drug gradients; live imaging	Cost, complexity, throughput limits	Pharmacokinetic modeling; precision-therapy screening

**Table 3.** Applications of organoid and spheroid models in hematologic malignancies. Representative 3D culture systems developed for acute myeloid leukemia, chronic myeloid leukemia, lymphoma, and multiple myeloma are summarized. Each model type incorporates disease-relevant stromal, immune, and vascular components to mimic the tumor microenvironment and to enable translational studies of therapy resistance, immune modulation, and precision drug screening.

Disease	Model Type	Key Components	Applications	Representative References
AML	Limited 3D co-culture or bone marrow organoid attempts	Mesenchymal stromal + endothelial + AML blasts	Proof-of-concept modeling of drug resistance and niche protection; early preclinical validation	[24–28,69–75,79,82]
CML	Experimental *ex vivo* stem cell expansion; microfluidic co-culture	Leukemic stem cells + stromal niche	LSC maintenance and early drug screening; no true organoids yet	[15–20,63,73,77,83,84]
Lymphoma	Lymphoid organoids or spheroid co-cultures	B/T cells + macrophages + stromal support	Immune checkpoint testing, drug screening, microenvironmental regulation	[40,41,43,47]
Multiple Myeloma	Bone marrow or myeloma organoids	Osteoblasts, osteoclasts, plasma cells, MSCs	Modeling bone lesions, IL-6-driven resistance, immune therapy testing	[29,42,44]
Anemia and Other Blood Disorders	Hematopoietic and yolk-sac-like 3D organoids	Erythroid progenitors + stromal + endothelial cells	Studying erythropoiesis and anemia pathophysiology	[31–34]
